# Editorial for: Bertoni et al. ex vivo fluorescence confocal microscopy: prostatic and periprostatic tissues atlas and evaluation of the learning curve

**DOI:** 10.1007/s00428-020-02763-2

**Published:** 2020-01-31

**Authors:** Till Braunschweig, Ruth Knüchel-Clarke

**Affiliations:** Institute of Pathology, University Clinic RWTH Aachen, 52062 Aachen, Germany

The work by Bertoni et al. in this issue of Virchows Archive presents a nice example of pathologists engaging in research to assess the quality and the reproducibility of a modern imaging technique that allows ex vivo assessment of fresh biopsies within only a few minutes. Although it is “only” a pilot study, the surgical pathologist most likely will be drawn to this paper curiously asking whether it can replace the tremendous and tedious manual work still carried out with frozen sections in our daily routine.

The commercial fluorescence confocal microscope (VivaScope® 2500 M-G4, NY, USA) used in this study has two different lasers that were both applied to create the picture, i.e., reflectance and fluorescence modes, and were used for each sample examination. The authors describe an examination depth of 200 μm, a vertical resolution of up to 4 μm, a magnification of × 550. If more time is applied for scanning, the penetration depth could be increased, however recent methods to our knowledge usually do not exceed 1000 μm. When ex vivo biopsies are used, image analysis can be carried out from at least two sides, increasing the depth of investigation and giving the option of three-dimensional reconstruction.

Besides the already commercially distributed instrument used here, there already is a remarkable number of other or related technologies tested on fresh tissue ex vivo mainly during surgery. One example is multiphoton microscopy with two photon-excited fluorescence by natural fluorophores and parallel second harmonic generation signals by fluorophores from the extracellular matrix. Further examples are light reflectance spectroscopy [1], optical coherence tomography (OCT) [2], high wavenumber spectra (so called Raman spectrometry) [3] and video-rate multi-colored structured illumination microscopy [4, 5], and also MRT-imaging with instruments that are in routine or research use for humans. An overview concerning alternative techniques to frozen sections is given by Eissa et al. last year [6].

The basic incentive for fast tissue analysis without significant processing is plausible as well for ex vivo and even more for in vivo applications. As pointed out by the reviewers of the paper, well-defined clinical indications are necessary which take into account the limitations of resolution and the time and costs involved. Ex vivo questions of resection margins put forward by the surgeons prevail, but sample validation for biorepository banks as well as, e.g., glomeruli detection in kidney biopsies would be apt areas of quality control, allowing short-term renewal of biopsy in case of insufficient findings.

The techniques of in vivo optical biopsies without any surgical interference is limited to a view from the surface (e.g., skin or mucosa) or lumen (e.g., vessels or urethra). Consequently, the dominant clinical field of application is dermatology, but ample work has also been carried out by gastroenterologists, urologists, ophthalmologists, pulmonologists, and in the field of head and neck tumor diagnosis. The methodologies are mostly applied for questions of precancerous lesions, early tumor invasion, or as with ex vivo procedures, tumor size and margins, especially taking into account the horizontal/lateral extension.

The value of a pathologist doing imaging research in cooperation with physicists, engineers, (bio-) chemists, and biologists has to be emphasized. Histology is the gold standard for tumor-related diagnosis and, to a lesser extent, major confirmatory instrument in inflammatory disease. The evaluation of the huge potential of imaging methods has to be assessed by the people providing this gold standard to put forward the questions that are relevant and avoid misdiagnosis. However, at the same time it needs the open mindedness that other methods than the old-fashioned histological techniques can provide sufficient or additional diagnostic answers. Some might also beat the accuracy of frozen sections in the diagnosis of cancer. Other disciplines as radiology with magnetic resonance tomography and nuclear medicine have been and are constantly refining the diagnostic accuracy, in part also using fluorescent tools for specific (tumor) tissue features as proliferation, metabolism, or specific molecules as, e.g., the prostate-specific membrane antigen (PSMA). In addition, pathology—as other imaging fields—increasingly integrates methods of artificial intelligence. While the insurmountable handcrafted glass tissue sections as the source for digital data is a hindrance in practicality, a method as presented here would allow digital pathology from fresh tissue and could not only be used for reproducibility studies amongst practicing pathologists but also as a source for further improvement of accuracy by deep learning algorithms including the relevant aspects of diagnosis.

Concluding, we are glad that this paper found its way into Virchows Archive, since in spite of the number of papers on digital histology on fresh tissue is steadily increasing since the beginning of the century, hardly any publication is found in a pathology journal. We suggest a higher contribution to the learning curve.
